# Identification of Expression Quantitative Trait Loci (eQTL) for Adipose-Specific Regulatory Mechanisms in Hanwoo (Korean Cattle)

**DOI:** 10.3390/ani15213082

**Published:** 2025-10-24

**Authors:** Junyoung Lee, Taejoon Jeong, Woncheoul Park, Sunsik Jang, Poong-Yeon Lee, Dajeong Lim

**Affiliations:** 1 Department of Animal Resources Science, College of Agriculture and Life Sciences, Chungnam National University, Daejeon 34134, Republic of Korea; dlwnsdud0714@naver.com (J.L.); jungtaejoon@gmail.com (T.J.); 2 Department of Bio-AI Convergence, Chungnam National University, Daejeon 34134, Republic of Korea; 3 National Institute of Animal Science, Rural Development Administration, Wanju 55365, Republic of Korea

**Keywords:** Hanwoo cattle, backfat, *cis*-eQTL, *trans*-eQTL

## Abstract

**Simple Summary:**

Improving beef quality in Hanwoo cattle requires a comprehensive understanding of the genetic regulation of fat accumulation in adipose tissue. In this study, we performed expression quantitative trait loci (eQTL) analysis using RNA-Seq data from backfat tissue of Hanwoo steers to identify regulatory variants influencing gene expression. We identified 5362 significant *cis*-eQTLs, including key genes associated with muscle development and fat accumulation. Several genomic loci were found to act as regulatory hotspots, controlling the expression of multiple genes. Notably, a variant at chromosome 21:17035557 emerged as a major hub where both *cis*- and *trans*-regulatory signals converge. These findings provide valuable insights into the regulatory architecture of adipose tissue in Hanwoo and offer molecular targets for precision breeding strategies aimed at enhancing meat quality.

**Abstract:**

Understanding the genetic regulatory mechanisms of fat accumulation is crucial for improving beef quality. Hanwoo (Korean native cattle) is renowned for its high intramuscular fat (marbling), yet the genetic regulation of adipose gene expression remains insufficiently understood. In this study, we performed expression quantitative trait loci (eQTL) analysis using RNA-Seq data and genotype data from backfat tissue of 75 Hanwoo steers to identify regulatory variants associated with adipose deposition. A total of 25,042 significant *cis*-eQTL associations (FDR < 0.05) were identified, and 5362 unique top *cis*-eQTL pairs were retained after gene-wise filtering. Key *cis*-regulated genes included *AGBL1*, *CACNG1*, *MYO18B*, and *DUSP29*, which are involved in cytoskeletal organization, muscle development and calcium signaling. Three major *cis*-regulatory hotspots were located on BTA15 (BTA15:50354741) and BTA21 (BTA21:21526143, and BTA21:21541921). Permutation-based analysis (100,000 iterations) was conducted to control false positives, identifying 12 statistically significant *trans*-eQTL hotspots (FDR q < 0.05), of which SNP 6:60512276 and SNP 21:17035557 exhibited extensive *trans*-regulatory activity influencing 429 and 161 genes, respectively. In particular, SNP 21:17035557 acted as a shared *cis*- and *trans*-regulatory hub, indicating hierarchical control of adipose gene networks. Functional enrichment analyses revealed significant involvement of cytoskeleton- and calcium-dependent pathways, highlighting the interplay between structural remodeling and metabolic regulation in adipose tissue. These findings provide a comprehensive, system-level view of adipose gene regulation in Hanwoo cattle and highlight candidate molecular targets for genome-assisted and precision breeding. Moreover, this study offers quantitative genomic resources that can support the development of prediction models and decision-support systems for improving carcass traits in Hanwoo breeding programs.

## 1. Introduction

Adipose tissue plays essential roles in mammals, including energy storage, hormonal regulation, and the maintenance of metabolic homeostasis [1]. In beef cattle, the anatomical distribution and development of fat depots are strongly associated with economically important carcass traits, such as marbling and subcutaneous fat thickness. While IMF is commonly linked to meat quality attributes such as tenderness, juiciness, and flavor, BFT serves as a key indicator of external fat coverage, yield grade, and overall carcass value in beef production systems [2].

Among adipose depots, subcutaneous backfat exhibits distinct characteristics compared with intramuscular and visceral fat. Transcriptomic and epigenomic analyses have revealed depot-specific variations in cellular lineage, lipid metabolism, insulin sensitivity, and immune cell infiltration, suggesting distinct, unique genetic and environmental regulation [3]. Derived primarily from mesodermal precursors, backfat displays elevated expression of genes associated with tissue architecture and contractile function [4]. These observations indicate that backfat is not merely an energy reservoir but plays active roles in tissue remodeling, intercellular signaling, and calcium-dependent metabolic processes. Therefore, elucidating its regulatory mechanisms is critical for understanding adipose distribution and carcass composition.

An expression quantitative trait locus (eQTL) is a genomic region that explains variation in gene expression levels among individuals. It typically involves genetic variants, such as single-nucleotide polymorphisms (SNPs), that regulate gene expression either locally (*cis*-eQTLs) or distantly (*trans*-eQTLs). By linking genetic variation to transcriptional output, eQTL analysis serves as a powerful approach for elucidating regulatory mechanisms and understanding how genotypic variation translates into complex phenotypic traits [5]. Recently, eQTL analysis has emerged as an indispensable tool in the livestock industry for dissecting the genetic architecture of economically important traits. It enables researchers to investigate how characteristics such as intramuscular fat (marbling), growth rate, and feed efficiency are genetically regulated [6]. Hanwoo cattle are characterized by their high intramuscular fat content and relatively slow growth rate, making them a valuable model for studying the genetic mechanisms underlying fat deposition. Despite their economic importance and distinct breed characteristics, studies on the gene regulatory architecture of Hanwoo remain limited compared to other beef cattle breeds. Expanding eQTL-based research in Hanwoo is therefore expected to provide critical insights into genetic improvement and contribute to enhancing the breed’s productivity and value.

At present, the genetic regulation of backfat among different adipose depots in cattle remains poorly understood. This knowledge gap largely reflects the fact that existing eQTL studies have predominantly focused on other tissues, such as the liver and *longissimus dorsi* muscle. Although backfat plays an important role in carcass grading, its distinct gene expression patterns have yet to be clearly defined. This represents a notable limitation, especially given that backfat exhibits unique transcriptomic signatures [7]. Collectively, these points highlight the need for eQTL studies specifically designed to capture the regulatory characteristics of individual adipose depots in cattle.

In Hanwoo cattle, recent studies have increasingly focused on identifying genetic factors associated with fat deposition and weight regulation by integrating eQTL mapping with gene expression profiling of specific tissues. Through these efforts, several key genes involved in lipid metabolism and adipocyte differentiation, such as *UBD*, *RGS2*, *FASN*, and *SCD*, have been identified [8]. Although related genes and metabolic pathways have been investigated across various tissues, the regulatory mechanisms operating within specific regions of subcutaneous fat that critically influence carcass yield remain poorly defined. Therefore, conducting eQTL analyses focused on backfat (BFT) to address this knowledge gap represents an urgent priority, as such research could ultimately elucidate the site-specific regulatory principles underlying fat deposition and adipose function in Hanwoo cattle.

Backfat tissue in Hanwoo cattle plays a pivotal role in determining carcass quality and economic value, yet its regulatory mechanisms remain insufficiently characterized. In this study, we investigated the genetic regulation of gene expression in backfat through expression quantitative trait locus (eQTL) analysis. By integrating RNA-Seq and genotype data from 75 animals, we combined transcriptional profiles with genomic variation to identify loci influencing adipose deposition. The functional relevance of these loci was further assessed through enrichment analyses of eQTL-associated genes, thereby linking regulatory variants to lipid metabolism and depot-specific biological pathways. This integrative approach not only enabled the detection of regulatory polymorphisms but also revealed their roles within the molecular networks underlying fat biology. Overall, our findings advance the understanding of adipose gene regulation in Hanwoo cattle and highlight potential genetic targets for precision breeding aimed at improving carcass quality and fat-related traits.

## 2. Materials and Methods

### 2.1. Animal and Tissue Collection

Backfat (BFT) tissue samples were collected post-mortem from 75 Hanwoo steers raised under standardized feeding and management conditions at the Hanwoo Cattle Research Institute, National Institute of Animal Science (NIAS), South Korea. Prior to slaughter, all animals were subjected to a 24 h feed withdrawal period under identical resting conditions to minimize physiological variation among individuals. Sampling was performed between August 2020 and May 2024, covering major growth stages from the growing to late fattening period. The age (mean ± SD, 14.3 ± 8.8 months) and body weight (mean ±SD, 356.9 ± 219.1 kg) of each animal were recorded at slaughter. All procedures were approved by the NIAS Animal Ethics Committee (approval no. NIAS20201979).

### 2.2. RNA Sequencing and Gene Expression Quantification

Total RNA was isolated from approximately 20 mg of backfat tissue using a standard commercial protocol combining Trizol lysis, DNase treatment, and column-based purification with QIAzol^®^ Lysis Reagent (Qiagen, Hilden, Germany) and the QIAamp^®^ DNA Mini Kit (Qiagen, Hilden, Germany). Extracted RNA was stored at −80 °C until library construction. RNA concentrations were measured using both a NanoDrop ND-1000 spectrophotometer (NanoDrop Technologies, Wilmington, DC, USA) and the Quant-iT™ RiboGreen RNA Assay Kit (Invitrogen, Carlsbad, CA, USA). RNA integrity and rRNA ratio were assessed with the Agilent TapeStation RNA ScreenTape system, and only samples meeting criteria of ≥0.5 µg total RNA, RIN ≥ 6.0, and rRNA ratio ≥ 1 were selected. RNA integrity was further confirmed with an Agilent Bioanalyzer (Agilent, Santa Clara, CA, USA), and only samples with RIN ≥ 8.0 were used for cDNA library preparation. These complementary quantification and integrity checks were performed sequentially to ensure sample quality rather than representing redundant steps.

RNA libraries were prepared from 0.5 µg of total RNA per sample using the Illumina TruSeq™ RNA Sample Preparation Kit (Illumina, San Diego, CA, USA) according to the manufacturer’s standard protocol. Libraries were sequenced on the Illumina NovaSeq 6000 platform, generating 100 bp paired-end reads with an average depth exceeding 6 Gb (~60 million reads per sample). Gene-level counts were obtained using featureCounts (subread v2.0.3) [9]. Lowly expressed genes (total read count < 200 or zero counts in ≥70 samples) were removed. Normalized expression values were obtained via variance stabilizing transformation (VST) using DESeq2 (v1.44.0; European Molecular Biology Laboratory, Heidelberg, Germany) [10], and the resulting expression matrix was used for downstream eQTL analysis. Sequencing data generated in this study have been deposited in the ArrayExpress database at the European Bioinformatics Institute (EMBL-EBI) under accession number E-MTAB-13398.

### 2.3. Genotype Data Preparation and Functional Annotation

Quality control of the RNA-Seq data was performed using FastQC (version 0.11.9; Babraham Bioinformatics, Cambridge, UK) [11] and summarized with MultiQC (version 1.27.1; Babraham Bioinformatics, Cambridge, UK) [12]. Preprocessing of the raw paired-end reads was conducted using Trimmomatic (version 0.39; Usadel Lab, Cambridge, UK) [13] with paired-end (PE) mode parameters (ILLUMINACLIP:TruSeq3-PE.fa:2:30:10 LEADING:3 TRAILING:3 SLIDINGWINDOW:4:15 MINLEN:36). Processed reads were aligned to the *Bos taurus* ARS-UCD1.3 reference genome using HISAT2 (version 2.2.1; Center for Computational Biology, Johns Hopkins University, Baltimore, MD, USA) [14], and the resulting alignments were converted to BAM format using SAMtools (version 1.13; Wellcome Sanger Institute, Cambridge, UK) [15]. Genomic variant discovery and refinement were conducted with the Genome Analysis Toolkit (GATK) (version 4.6.1.0; Broad Institute, Cambridge, MA, USA) [16]. PCR duplicate reads were removed with Picard MarkDuplicates, and spliced RNA-seq alignments were processed using GATK SplitNCigarReads to handle reads spanning exon-exon junctions. Variant calling was performed with GATK HaplotypeCaller in GVCF mode, followed by joint genotyping using GenotypeGVCFs. Variant quality control was applied using VariantFiltration with the following thresholds: QD < 2.0, FS > 60.0, and MQ < 40.0, retaining only high-quality variants flagged as PASS. Single-nucleotide polymorphisms (SNPs) were further filtered for a minor allele frequency (MAF) > 0.01 and subjected to linkage disequilibrium (LD) pruning (r^2^ < 0.2) using PLINK (version 1.90b6.24; Broad Institute, Cambridge, MA, USA) [17]. Only SNPs located within or near expressed genes were retained for downstream eQTL analysis. To predict the potential functional effects of the identified variants, the filtered VCF file was annotated using SnpEff (version 5.2; Johns Hopkins University, Baltimore, MD, USA) [18], based on the *Bos taurus* ARS-UCD1.3.105 reference genome database.

### 2.4. Identification of eQTLs and Regulatory Hotspot Mapping

The association between SNP genotypes and gene expression levels was evaluated using the Matrix eQTL package (version 2.3; University of North Carolina, Chapel Hill, NC, USA) [19]. An additive linear model was employed, with hard-called genotypes coded as 0, 1, or 2 copies of the effect (minor) allele (useModel = modelLINEAR). The analysis incorporated the genotype matrix, normalized expression data, age as a covariate, and genomic coordinates of genes and variants. *Cis*-eQTLs were defined as SNP-gene pairs located within ±100 kb of the corresponding gene, while *trans*-eQTLs were defined as pairs exceeding this distance. The significance thresholds were set at *p* < 0.02 for *cis*-eQTLs and *p* < 1 × 10^−6^ for *trans*-eQTLs. Residual errors were assumed to be independent across samples (*errorCovariance = numeric()*), and cis and trans associations were analyzed separately. Statistically significant eQTLs were identified at a false discovery rate (FDR) ≤ 0.05. To assess genotype-dependent variation in gene expression, normalized expression levels were compared across genotype groups. Significant *cis*-eQTL results (FDR < 0.05) were further processed using the dplyr package (version 1.1.4; RStudio, Boston, USA) in R. For each gene, duplicate entries were removed by retaining only the top-associated SNP with the lowest FDR value. Functional annotation was performed by mapping Ensembl gene IDs to corresponding gene symbols and names using the biomaRt package (version 2.60.1; European Bioinformatics Institute, Cambridge, UK).

An expression quantitative trait locus (eQTL) hotspot is generally defined as a genomic locus that regulates the expression of multiple genes [20]. Following this concept, *cis*-eQTL hotspots were defined as SNPs associated with seven or more unique genes (n ≥ 7), based on the distribution of *cis*-regulated genes per SNP. This threshold was chosen in accordance with a previous study [21]. To identify *trans*-eQTL hotspots, the number of unique genes significantly regulated by each SNP was calculated from the Matrix eQTL results. The distribution of regulated gene counts per SNP was highly skewed, with the majority of SNPs influencing only a few genes, whereas a small subset affected the expression of hundreds of distant genes [22]. Accordingly, *trans*-eQTL hotspots were defined as SNPs whose number of regulated genes exceeded the 99th percentile of this distribution. A similar quantitative cutoff has been reported previously, where hotspots were defined as genomic bins containing more than 12 eQTLs [23], supporting the rationale of our quantile-based threshold. This percentile-based approach identifies a limited number of SNPs acting as long-range regulatory hubs that control the expression of multiple genes, thereby enabling the detection of biologically meaningful hotspot candidates. Applying a stringent cutoff minimizes false positives and ensures that only loci with strong regulatory potential are retained. To estimate the false-positive rate of the *trans*-eQTL hotspots, a permutation-based analysis was performed after hotspot identification. Specifically, the data were randomly permuted 100,000 times to evaluate the false-positive rate for each *trans*-eQTL signal [24].

### 2.5. Functional Enrichment Analysis Using ClueGO

To investigate the biological functions of genes associated with significant *cis*-eQTLs in Hanwoo backfat tissue, functional enrichment analysis was conducted using the ClueGO plugin (version 2.5.9; Institute of Genetics and Molecular and Cellular Biology, Strasbourg, France) [25] implemented in Cytoscape (version 3.10.3; Cytoscape Consortium, San Diego, CA, USA) [26]. A kappa score threshold of 0.4, the default parameter in ClueGO, was applied to cluster functionally related terms into coherent groups. The resulting enrichment network was visualized as a color-coded graph, where each node represented a distinct biological theme.

## 3. Results

### 3.1. RNA-Seq Data Processing and Variant Calling for eQTL Analysis

To investigate the genetic basis underlying gene expression variation in Hanwoo backfat tissue (BFT), we generated RNA-Seq data from 75 BFT samples. On average, each sample yielded approximately 35.1 million paired-end reads (ranging from 25.7 M to 43.8 M), with an overall alignment rate of 97.7% (range: 90.2–99.2%) to the *Bos taurus* reference genome (ARS-UCD1.3) using HISAT2 [27]. From the initial 5,290,243 SNPs, we retained only variants labeled as “PASS” (resulting in 5,256,746 variants), removed those with a minor allele frequency (MAF) below 0.01, and conducted linkage disequilibrium (LD) pruning with an r^2^ threshold of 0.2. After quality control, a total of 2,455,435 high-confidence SNPs were retained for eQTL analysis. Gene expression was quantified using featureCounts. Genes with fewer than 200 total counts or with zero counts in more than 70 samples were excluded to minimize noise from lowly expressed features. The remaining 22,897 genes were transformed using the variance-stabilizing transformation (vst) function implemented in the DESeq2 package and subsequently used for downstream analyses.

### 3.2. eQTL Mapping in Backfat Tissue

**Figure 1:**
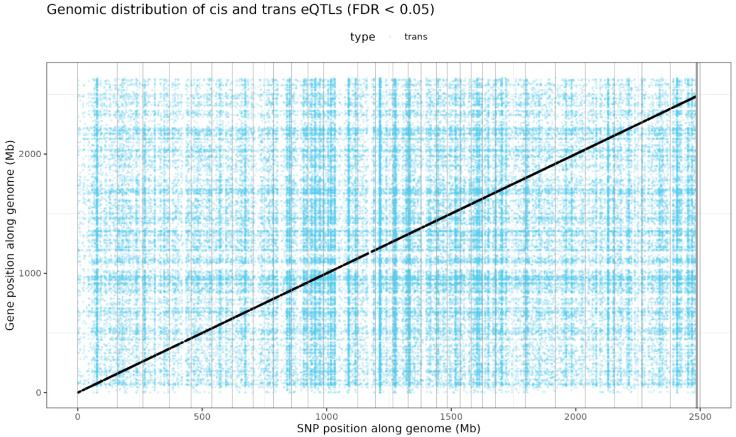
Genomic distribution of significant *cis*- and *trans*-eQTLs (FDR < 0.05). The x-axis shows SNP positions, and the y-axis shows the positions of their associated genes. Each dot represents an SNP-gene pair. The black diagonal indicates positional correspondence for cis associations; points within ±100 kb of the line represent *cis*-eQTLs, while those farther away represent *trans*-eQTLs.

**Figure 2:**
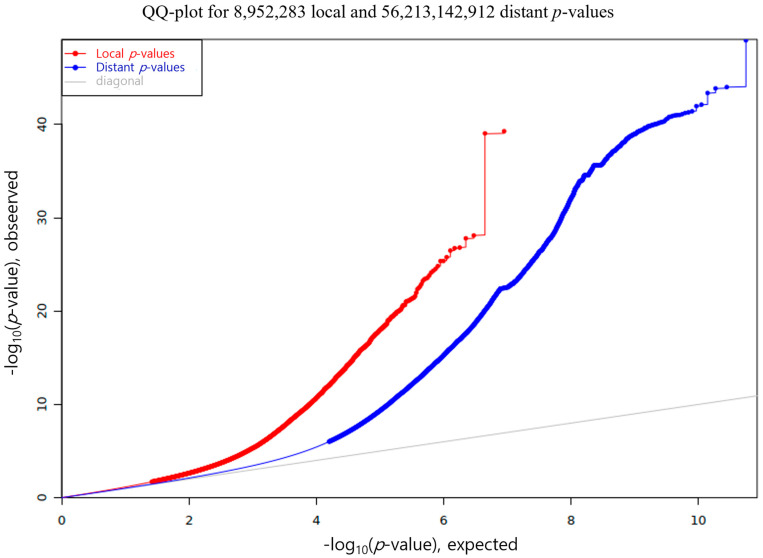
Quantile-quantile (QQ) plot comparing observed versus expected *p*-values for *cis* and *trans* eQTLs in Hanwoo backfat tissue. The red curve represents 8,952,283 *cis* (local) SNP-gene pairs, and the blue curve represents 56,213,142,912 *trans* (distant) SNP-gene pairs. The grey diagonal line indicates the null hypothesis of no association. Deviations above the diagonal reflect enrichment of significant associations beyond those expected by chance.

Genotype-expression associations were evaluated using the linear model implemented in Matrix eQTL software (version 2.3; University of North Carolina, Chapel Hill, NC, USA), with age included as a covariate. Given their higher reproducibility, interpretability, and stronger likelihood of direct regulatory influence, *cis*-eQTLs were prioritized for analysis. *Cis*-eQTLs were defined as SNP-gene pairs located within 100 kb of each other [28]. As a result, we identified a total of 25,042 statistically significant *cis*-eQTLs at a FDR of less than 0.05 [29] (Figure 1). To streamline downstream interpretation, we retained only the most significant *cis*-eQTLs per gene, yielding a subset of 5362 unique top *cis*-eQTL pairs. This gene-wise filtering enabled the prioritization of regulatory variants exerting the strongest effects on gene expression, thereby enhancing functional interpretation and pathway analysis. To assess the overall distribution of statistical significance, we constructed a quantile-quantile (QQ) plot comparing observed and expected *p*-values for local (*cis*) and distant (*trans*) associations. The local eQTLs displayed a pronounced deviation from the null distribution, indicating the presence of strong and widespread *cis*-regulatory effects in Hanwoo backfat tissue (Figure 2).

### 3.3. Identification of Significant cis-eQTL Associations and cis-eQTL Hotspot

**Figure 3:**
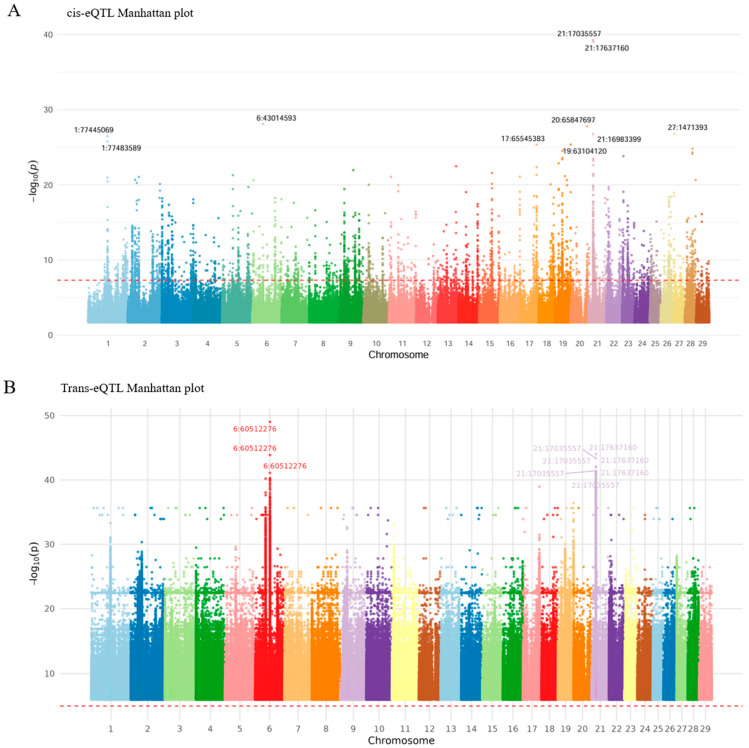
Manhattan plots of (**A**) *cis*-eQTL and (**B**) *trans*-eQTL associations in Hanwoo backfat tissue. Each dot represents an SNP-gene pair, with genomic position on the x-axis and statistical significance (−log_10_P) on the y-axis. The red dashed line denotes the genome-wide significance threshold (P = 5 × 10^−8^). In (**A**), *cis*-eQTL signals are distributed across all autosomes, and the ten most significant SNPs are labeled using the chromosome:position notation. In (**B**), *trans*-eQTL signals are likewise distributed genome-wide, with the ten most significant SNPs labeled in the same notation.

**Figure 4:**
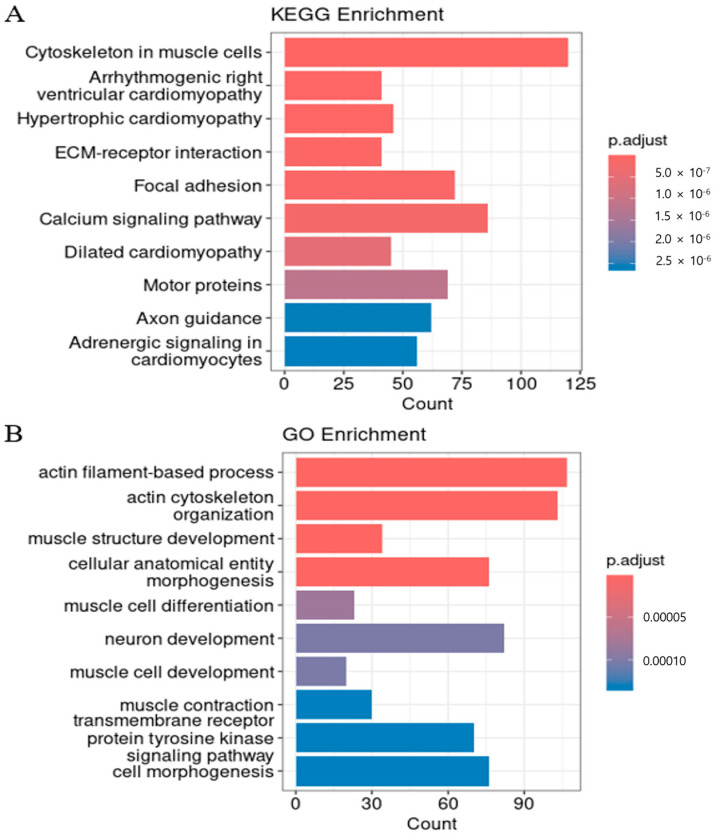
Functional enrichment analysis of *cis*-eQTL-associated genes in Hanwoo backfat tissue. (**A**) KEGG pathway enrichment. (**B**) Gene Ontology (GO) Biological Process enrichment. The x-axis indicates the number of genes (gene count) in each term, while the color gradient reflects the adjusted *p*-value, with deeper colors indicating stronger statistical significance.

Among the significant *cis*-eQTLs identified, the top ten SNP-gene pairs ranked by *p*-value are summarized in Table 1. These associations exhibited extremely low *p*-values (e.g., 5.25 × 10^−40^), strong effect sizes, and moderate-to-high minor allele frequencies (MAFs). To further characterize their potential functional impact, the consequences of each SNP were annotated using the SnpEff software (version 5.2; Johns Hopkins University, Baltimore, USA). SnpEff classifies SNPs according to their genic context, including intron variants, synonymous variants, 5′ UTR variants, 3′ UTR variants, upstream and downstream gene variants, splice region variants, missense variants, intergenic regions, and non-coding transcript exon variants. The complete list of significant *cis*-eQTLs is provided in Supplementary Table S1, which also contains detailed statistics such as β ± SE, effect direction, *t*-statistic, explained variance, and 95% confidence intervals (CI95) for each SNP-gene pair. The explained variance for each eGene is additionally summarized in Supplementary Table S4, where the lead SNP per gene and its corresponding statistics are reported. Figure 3 depicts the genomic distribution of the 25,042 significant *cis*-eQTL associations (within 100 kb), highlighting strong local gene expression regulatory effects in Hanwoo backfat tissue. To investigate the underlying regulatory architecture, we defined SNPs associated with seven or more genes (*n* ≥ 7) as *cis*-eQTL hotspots. Consequently, SNPs located at BTA15:50354741, BTA21:21526143, and BTA21:21541921 were identified as hotspots. Each of these SNPs displayed strong associations with seven distinct target genes, supporting their roles as key regulatory hubs (Figure 3A). Functional enrichment analysis was also performed to characterize the biological functions of the cis-eQTL-associated genes (Figure 4).

### 3.4. Functional Characterization of Genes Regulated by Cis-eQTL Genes

**Figure 5:**
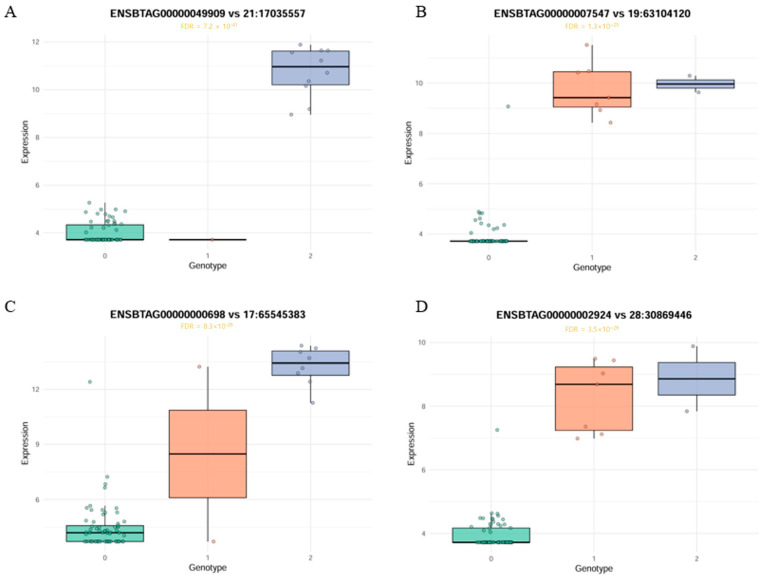
Boxplots showing *cis*-eQTL effects for the four most significant genes in Hanwoo backfat tissue. Gene expression levels are stratified by SNP genotype (0, 1, 2), indicating significant allele-specific regulatory effects. FDR-adjusted *p*-values are shown for each association. (**A**) *AGBL1* at 21:17035557, (**B**) *CACNG1* at 19:63104120, (**C**) *MYO18B* at 17:65545383, (**D**) *DUSP29* at 28:30869446.

**Figure 6:**
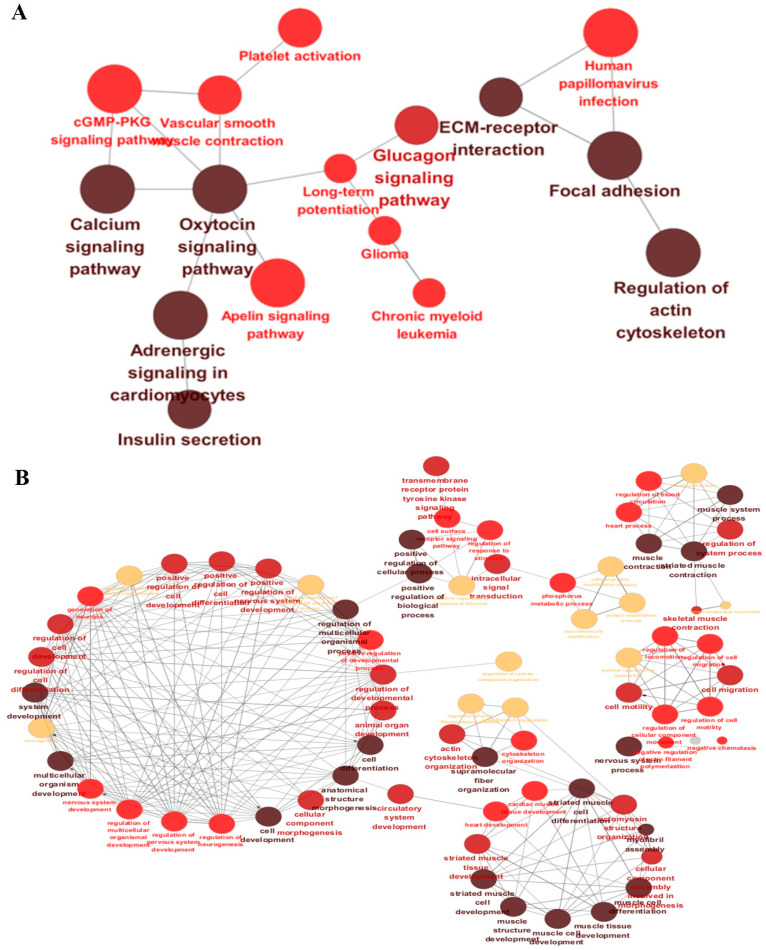
Network visualization of enriched functional categories among *cis*-eQTL-associated genes in Hanwoo backfat tissue. (**A**) KEGG pathway enrichment network. Each node represents a KEGG pathway, with edges connecting pathways that share genes. Node colors indicate functional clusters, with the same color denoting related pathways. (**B**) GO Biological Process enrichment network. Each node represents a significantly enriched GO term, with edges indicating shared gene content. Node colors indicate functional themes, and only clustered GO terms are shown.

Gene expression patterns for the top four genes associated with *cis*-eQTLs were visualized as representative examples of allele-specific regulatory effects (Figure 5). *AGBL1*, *CACNG1*, *MYO18B*, and *DUSP29* genes displayed distinct gene expression patterns across SNP genotypes (0 = homozygous reference, 1 = heterozygous, 2 = homozygous alternative), indicating allele dosage effects. Gene expression levels were highly significantly associated with SNP genotypes, with FDR-corrected *p*-values ranging from 1.5 × 10^−25^ to 5.2 × 10^−40^. Expression of the *AGBL1* gene was significantly higher in individuals with genotype 2, suggesting an additive regulatory effect in which expression increases with allele dosage. The expression of the *CACNG1* gene was highest in individuals with genotype 1, decreased slightly in 2, and was almost absent in 0. This non-linear expression pattern suggests the possibility of a non-additive regulatory effect, specifically a heterozygous overdominance or dominance effect. *MYO18B* expression was highest in genotype 2, intermediate in genotype 1, and lowest in genotype 0, indicating an additive dosage effect. Similarly, *DUSP29* expression increased steadily from genotype 0 to genotype 2, supporting an additive regulatory mechanism with allele number. KEGG and GO enrichment of *cis*-eQTL genes in Hanwoo backfat identified 282 significant terms after Bonferroni correction (*p* < 0.05), forming a functional network (Figure 6A). To enhance interpretability, kappa score-based clustering was applied using a threshold of 0.4, a commonly adopted cutoff that balances specificity and connectivity [30]. Gray and isolated nodes lacking sufficient similarity were excluded, following the standard ClueGO workflow [25]. Similarly, GO term enrichment analysis (Bonferroni-adjusted *p* < 0.05) revealed major clusters related to muscle tissue development, immune responses, and lipid metabolism (Figure 6B). To complement these network visualizations, representative GO terms with their associated genes, FDR, and *p*-values are summarized in Table 2.

### 3.5. Identification and Functional Enrichment Analysis of a trans-eQTL Hotspot

To identify genomic regions exhibiting extensive distant regulatory activity, we quantified the number of unique genes significantly regulated by each SNP based on the Matrix eQTL results. The 99th percentile of the distribution corresponded to ten regulated genes per SNP, which was used as the cutoff to define extensive regulatory hotspots. Accordingly, we identified 147 SNPs regulating more than ten genes as *trans*-eQTL hotspots (Supplementary Table S2). Among these, SNP 6:60512276 exhibited the strongest *trans*-regulatory effect, influencing the expression of 429 genes. SNP 21:17035557 regulated 161 genes and simultaneously displayed a strong *cis*-eQTL signal, suggesting that this locus may act as a shared regulatory hub where *cis*- and *trans*-regulatory mechanisms converge.

To further evaluate the statistical significance of the identified *trans*-eQTL hotspots, we performed permutation analyses with 100,000 iterations to estimate the false discovery rate (FDR). This analysis confirmed the robustness of the detected signals (FDR q-value < 0.05), identifying 12 statistically significant *trans*-eQTL hotspots, which are summarized in Supplementary Table S5. Among them, SNP 6:60512276 exhibited the most significant association (*p*-value = 0.00001, FDR q-value = 0.000735), underscoring its role as a major *trans*-regulatory hub. In contrast, SNP 21:17035557 displayed both strong *cis*- and *trans*-eQTL signals (*p*-value = 0.00002, FDR q-value = 0.000735), suggesting that this locus functions as a genuine genetic master regulator where *cis*- and *trans*-regulatory signals converge.

**Figure 7:**
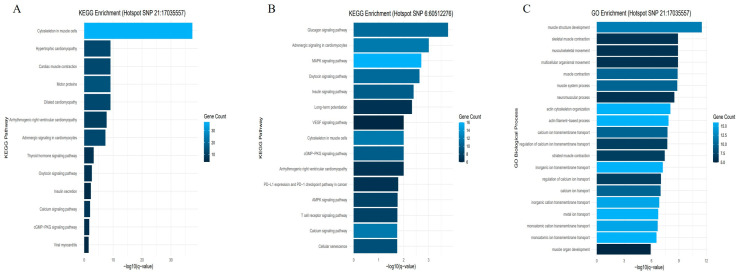
KEGG and GO enrichment analyses of genes regulated by the top *trans*-eQTL hotspots in Hanwoo backfat tissue. KEGG and GO enrichment analyses were performed for genes regulated by the two most significant *trans*-eQTL hotspots, SNP 6:60512276 and SNP 21:17035557. Panel (**A**) shows the KEGG pathway analysis for SNP 21:17035557, panel (**B**) shows the KEGG pathway analysis for SNP 6:60512276, and panel (**C**) presents the GO Biological Process analysis for genes regulated by SNP 21:17035557.

To investigate the biological and functional significance of the *trans*-eQTL hotspots, we performed enrichment analyses on the target genes regulated by SNPs within these hotspots (Figure 7). For SNP 6:60512276, significant KEGG pathway enrichment was observed in the Cytoskeleton in muscle cells pathway (bta04820, *p* = 3.84 × 10^−4^) and the Calcium signaling pathway (bta04020, *p* = 1.20 × 10^−3^). Gene Ontology (GO) analysis further identified the ubiquitin-dependent protein catabolic process (GO:0006511, *p* = 1.45 × 10^−4^) as a significantly enriched biological process. Likewise, KEGG pathway analysis for SNP 21:17035557 revealed strong enrichment in the Cytoskeleton in muscle cells pathway (bta04820, *p* = 3.53 × 10^−40^) and in the Calcium signaling pathway (bta04020, *p* = 1.02 × 10^−3^). In addition, GO enrichment highlighted multiple biological processes associated with muscle structure development, contraction, cytoskeletal organization, and calcium ion homeostasis.

## 4. Discussion

The genomic distribution of eQTLs revealed a predominance of local regulatory effects in Hanwoo backfat tissue (Figure 1). Most significant SNP-gene pairs were clustered along the diagonal, indicating that *cis*-eQTLs tend to occur near their corresponding target genes. This enrichment supports the principle that *cis*-regulatory variants reside within promoters, enhancers, or untranslated regions (UTRs) controlling transcription [31]. The broad coverage of *cis*-eQTLs further suggests strong local genetic regulation of adipose gene expression [28]. In contrast, SNP-gene pairs located away from the diagonal reflect the complexity of *trans*-regulation, emphasizing the need for larger sample sizes or multi-tissue analyses to fully capture distant regulatory effects. Similarly, the quantile-quantile (QQ) plot in Figure 2 illustrates this trend. A total of 8,952,283 SNP-gene pairs within ±100 kb were tested in the *cis*-eQTL analysis (red line), and clear deviations from the expected null distribution (gray line) were observed. These deviations indicate that only a small fraction of tested pairs exhibit true *cis*-regulatory associations, strongly suggesting the presence of robust genetic signals influencing gene expression beyond what would be expected by chance.

Figure 3 presents the genome-wide distribution of *cis*- and *trans*-eQTLs identified in Hanwoo backfat tissue. Significant *cis*-eQTL signals (*p* < 5 × 10^−8^) were evenly distributed across all 29 autosomes, with a dense cluster observed on chromosome BTA21 at position 17,035,557 (Figure 3A). This region appears to serve as a local regulatory hotspot concentrating multiple *cis*-regulatory effects. The *trans*-eQTL analysis revealed that certain SNPs were significantly associated with a large number of distant genes. Notably, SNP 6:60512276 showed strong associations with 429 *trans*-genes, suggesting its potential role as a long-range regulatory hub (Figure 3B). Interestingly, some *trans*-eQTL SNPs were located at the same genomic positions as *cis*-eQTLs. For instance, BTA21:17035557 functioned as both a *cis*- and *trans*-eQTL, being associated with 161 distant genes. Among these, we identified key *cis*-regulated genes such as *CACNG1* and *MYO18B*, which displayed particularly strong *cis*-eQTL signals. These findings suggest that a single genomic region can exert both local and distal regulatory influences, simultaneously affecting nearby and distant genes.

Previous studies have reported that eQTL hotspots frequently overlap with histone modification marks and open chromatin regions, underscoring their functional roles in gene regulation [28]. These findings suggest that the locus at BTA21:17035557 may serve as a central regulatory hub influencing gene expression in backfat tissue, potentially through transcriptional or chromatin-level mechanisms.

KEGG pathway enrichment analysis of 3770 *cis*-eQTL-associated genes revealed significant overrepresentation of pathways related to muscle structure and signaling (Figure 4A). Among these, the “Cytoskeleton in muscle cells” pathway (bta04820, *p* = 1.78 × 10^−32^) showed the highest statistical significance, suggesting that genetic variation within adipose tissue, such as Hanwoo backfat, may influence the expression of genes involved in structural stability and contractile function. Representative *cis*-eGenes included *MYH7*, *ACTN2*, *ANK3*, *ITGA7*, and *MYOM1/2/3*. These findings align with recent molecular biology studies [32] demonstrating that adipocytes can exhibit muscle-like gene expression patterns and structural characteristics under specific physiological conditions. For instance, the acetylation level of α-tubulin—a key component of microtubules—was reported to increase markedly during adipogenesis, the process of fat cell differentiation, whereas disruption of α-tubulin acetylation significantly inhibited adipocyte differentiation [33]. Although canonical enzymes responsible for this modification were not detected among the leading cis signals, *ANK3* provides a potential mechanistic link; in neuronal models, its repression has been shown to reduce α-tubulin acetylation and destabilize microtubules [34]. Collectively, these results suggest that cytoskeleton-related pathways annotated as muscle-specific also play important roles in adipose tissue. Consistently, GO enrichment analysis further highlighted pathways associated with cytoskeletal remodeling, muscle development, and signaling regulation (Table 2), including actin filament organization, sarcomere assembly, focal adhesion, and receptor tyrosine kinase signaling, indicating that *cis*-eQTLs contribute to both structural stability and intracellular signaling within adipose tissue.

The calcium signaling (bta04020, *p* = 3.38 × 10^−9^) and focal adhesion (bta04510, *p* = 1.39 × 10^−9^) pathways indicate that *cis*-eQTLs regulate genes involved in intracellular signaling and cell-matrix interactions—processes that are central to adipocyte differentiation, lipid storage, and responses to mechanical stimuli [35]. Previous studies have also reported enrichment of calcium ion-related pathways associated with traits such as meat tenderness, feed efficiency, and muscle contraction [36]. Furthermore, enrichment of ECM-receptor interaction (bta04512, *p* = 6.92 × 10^−10^) and motor protein (bta04814, *p* = 2.83 × 10^−8^) pathways further emphasizes the importance of cytoskeletal remodeling and associated signaling in bovine backfat development [37]. Collectively, these results highlight that cytoskeletal and ECM signaling are essential for adipocyte differentiation and structural stability, influencing both the metabolic and mechanical properties of adipose tissue.

Functional enrichment analysis in the GO Biological Process category identified several significantly overrepresented terms, including muscle structure development (GO:0061061, *p* = 3.57 × 10^−10^), actin filament organization (GO:0007015, *p* = 5.98 × 10^−6^), striated muscle cell development (GO:0055002, *p* = 8.86 × 10^−6^), and cellular component assembly involved in morphogenesis (GO:0010927, *p* = 1.68 × 10^−5^) (Figure 4B). These findings indicate that cytoskeletal remodeling, structural regulation, and intracellular signaling play pivotal roles in adipocyte differentiation, suggesting that *cis*-eQTLs may influence backfat tissue function through modulation of these pathways. Notably, cytoskeletal and extracellular matrix (ECM) signaling are already well established as key regulators of cell shape changes, lipid accumulation, and depot-specific metabolic activation in adipose biology [38]. Moreover, the enrichment of broad regulatory terms such as positive regulation of biological processes suggests that *cis*-eQTLs may contribute to transcriptional activation during adipocyte development, thereby affecting tissue architecture and fat-related phenotypic traits [39].

As shown in Table 1, many of the top *cis*-eQTLs were located within intronic regions. This finding is consistent with previous studies reporting that a substantial proportion of *cis*-eQTLs are positioned within introns [40]. Intronic variants identified as significant in our eQTL analysis have been linked to transcriptional regulatory mechanisms [41]. Although such variants were once considered non-functional, accumulating evidence now indicates that intronic variants can participate in diverse modes of gene regulation. These observations suggest that the muscle development-related genes identified in this study may be finely modulated through intronic regulatory mechanisms rather than solely at the transcript abundance level.

*AGBL1*, one of the top *cis*-eQTL genes, encodes a carboxypeptidase that regulates microtubule remodeling via tubulin deglutamylation, a process essential for cytoskeletal stability and cell morphology [42]. Beyond this role, *AGBL1* has been implicated in lipid accumulation and adipocyte differentiation [43]. CACNG1, a member of the CACNG1-8 family, encodes auxiliary subunits of voltage-dependent calcium channels that mediate calcium influx in excitable cells [44]. Recently, it was identified as a skeletal muscle-specific gene in buffalo, exhibiting strong promoter activity [45]. The presence of a *cis*-eQTL signal for *CACNG1* in Hanwoo backfat tissue suggests a potential tissue-specific regulatory mechanism in adipose cells. *MYO18B* (myosin XVIIIB), a non-motor myosin, is required for sarcomere integrity. In zebrafish, complete loss of *MYO18B* disrupts sarcomere formation, whereas partial loss impairs filament alignment [46]. *MYO18B* exhibited a strong *cis*-eQTL signal in Hanwoo backfat tissue. Enrichment analysis highlighted processes such as actin filament-based organization (GO:0030029, *p* = 1.07 × 10^−10^), indicating that transcriptional regulation of structural genes remains active in adipose tissue. *DUSP29*, a dual-specificity phosphatase, regulates skeletal muscle homeostasis through the MAPK, AMPK, and glucocorticoid receptor pathways [47]. In Nellore cattle, *DUSP29* has been proposed as a candidate gene for carcass traits due to its role in muscle differentiation [48]. Although *AGBL1*, *CACNG1*, *MYO18B*, and *DUSP29* are not typically classified as adipose tissue-specific genes and are primarily associated with muscle structure, signaling, or development, their strong *cis*-eQTL signals in Hanwoo backfat suggest that they may exert regulatory functions beyond their canonical roles. This pattern may reflect the shared mesodermal origin and developmental plasticity between muscle and adipose tissues [49] or imply that even low-level expression of muscle-associated genes in adipose tissue can be biologically meaningful and subject to genetic regulation [50].

BTA21:21526143 and BTA21:21541921 were identified as *cis*-eQTL hotspots regulating seven genes. Among these, only *IDH2* had an assigned gene symbol, while the remaining six genes remain unannotated. Therefore, we focused our analysis on *IDH2* to interpret the biological function of this hotspot. *IDH2* encodes a mitochondrial enzyme involved in the tricarboxylic acid (TCA) cycle and plays a critical role in NADPH production, antioxidant defense, and metabolic regulation [51]. It may act as an integrative metabolic regulator, contributing to fat storage, oxidative stress response, and cellular differentiation. The remaining six genes, currently represented only by ENSEMBL IDs without functional annotation, were also significantly associated with the same *cis*-eQTLs and exhibited differential expression. This suggests that they may share similar metabolic or regulatory roles with *IDH2*. BTA15:50354741 was found to regulate genes belonging to the olfactory receptor family 51. Although classically associated with olfactory functions, *OR51E2* is also expressed in non-olfactory tissues such as the intestine and adipose tissue, where it has been implicated in diverse physiological processes, including fat metabolism, cell differentiation, and cancer biology [52]. These findings indicate that genes associated with fat metabolism are not only enriched within *cis*-eQTL hotspots but also participate in multiple metabolic and developmental pathways relevant to adipose function.

To further elucidate the gene expression regulatory architecture in Hanwoo backfat tissue, we investigated the potential for long-range genetic regulation (*trans*-eQTLs) beyond the effects of local regulatory variants (*cis*-eQTLs). Previous studies in mice [53], maize [54], and humans [55] have demonstrated that certain genomic regions, known as *trans*-eQTL hotspots, can broadly regulate the expression of numerous distant genes through master regulatory factors such as transcription factors or chromatin modifiers. Given that fat accumulation is a multifactorial process involving signal transduction, energy homeostasis, and tissue structural remodeling, we hypothesized that similar regulatory hubs might exist in the backfat tissue of Hanwoo cattle. Our analysis identified 147 *trans*-eQTL hotspots, among which 12 were confirmed as statistically significant *trans*-regulatory signals based on permutation testing (FDR Q < 0.05). Among them, SNP 6:60512276 regulated 429 genes, representing the largest hotspot, while SNP 21:17035557 regulated 161 genes, forming another major hotspot. Genes associated with hotspot 6:60512276 were significantly enriched in the cytoskeleton (bta04820, *p* = 3.84 × 10^−4^), calcium signaling (bta04020, *p* = 1.20 × 10^−3^), and energy metabolism pathways. The overlap between *cis*- and *trans*-eQTL results suggests that this hotspot functions as a *trans*-regulatory element influencing muscle organization and intracellular signaling. This region, therefore, appears central to adipocyte structure, metabolism, and intercellular communication. Similarly, SNP 21:17035557 was linked to the cytoskeleton (bta04820, *p* = 3.53 × 10^−40^) and calcium signaling (bta04020, *p* = 1.02 × 10^−3^) pathways, consistent with *cis*-eQTL findings. It also influenced *cis*-regulated genes such as *CACNG1* and *MYO18B*, which ranked among the most significant associations in the *cis*-eQTL analysis. The coexistence of *cis*- and *trans*-regulated clusters supports a hierarchical regulatory architecture in which local and global regulators cooperatively orchestrate pathways governing fat accumulation and meat quality. This region may thus harbor master regulatory genes that shape transcriptional programs underlying adipose deposition and tissue development. The full list of genes regulated by SNPs 6:60512276 and 21:17035557 is provided in Supplementary Table S3.

## 5. Conclusions

We conducted both *cis*- and *trans*-eQTL analyses in Hanwoo backfat tissue to elucidate the genetic networks underlying fat-related traits. Key *cis*-eQTL genes (*AGBL1*, *CACNG1*, *MYO18B*, and *DUSP29*) showed strong local associations and were primarily linked to cytoskeletal organization and muscle development. In addition, we identified 147 *trans*-eQTL hotspots, among which SNP 21:17035557 was significant in both *cis* and *trans* analyses, suggesting the presence of a shared regulatory hub. Collectively, these findings demonstrate that adipose gene expression in Hanwoo cattle is governed by a hierarchical regulatory architecture involving both proximal (*cis*) and distal (*trans*) elements. The resulting integrative networks provide a systems-level understanding of fat metabolism and offer a foundation for genome-informed selection and prediction strategies to improve carcass and meat quality traits in Hanwoo cattle.

## Figures and Tables

**Table 1:** Top 10 significant *cis*-eQTL SNP-gene pairs identified through Matrix eQTL analysis, ranked by *p*-value. For each gene, only the most significant SNP was selected as the representative *cis*-eQTL (FDR < 0.05). The table includes the chromosome number (CHR), genomic position (POS), combined SNP ID (CHR:POS), Ensembl gene ID, *p*-value, FDR, gene symbol (if available), and functional annotation.

Rank	CHR	POS	SNP	Gene ID	*p*-Value	FDR	Beta	MAF	Symbol	Variant Type
1	21	17035557	21:17035557	ENSBTAG00000049909	5.25 x 10^−40^	4 × 10^−33^	3.264562	0.14	*AGBL1*	Intron variant
2	6	43014593	6:43014593	ENSBTAG00000067097	7.92 × 10^−29^	2.36 × 10^−22^	2.255543	0.113333	NA	Intron variant
3	20	65847697	20:65847697	ENSBTAG00000051967	1.61 × 10^−28^	3.6 × 10^−22^	2.54634	0.02	NA	Upstream gene variant
4	27	1471393	27:1471393	ENSBTAG00000009387	1.58 × 10^−27^	2.71 × 10^−21^	4.559134	0.173333	*MYOM2*	Intron variant
5	1	77445069	1:77445069	ENSBTAG00000015460	3.3 × 10^−27^	4.23 × 10^−21^	2.994177	0.113333	*TP63*	Intron variant
6	19	63104120	19:63104120	ENSBTAG00000007547	4.37 × 10^−26^	3.98 × 10^−20^	4.236663	0.073333	*CACNG1*	Intron variant
7	17	65545383	17:65545383	ENSBTAG00000000698	4.44 × 10^−26^	3.98 × 10^−20^	4.30032	0.12	*MYO18B*	Intron variant
8	28	30869446	28:30869446	ENSBTAG00000002924	1.54 × 10^−25^	1.25 × 10^−19^	3.264954	0.073333	*DUSP29*	Synonymous variant
9	19	29483289	19:29483289	ENSBTAG00000009702	3.15 × 10^−25^	2.35 × 10^−19^	2.823308	0.16	*MYH8*	Splice region & Intron variant
10	28	30869446	28:30869446	ENSBTAG00000059987	4.95 × 10^−25^	3.41 × 10^−19^	3.610908	0.073333	*DUSP13A*	Synonymous variant

*p*-values, effect sizes (Beta), and false discovery rate (FDR) values were obtained from the Matrix eQTL linear regression analysis. Beta represents the estimated allelic effect size, indicating the change in normalized gene expression per allele dosage. The FDR was calculated within Matrix eQTL using the Benjamini-Hochberg correction to control for multiple testing.

**Table 2:** Representative GO terms enriched among *cis*-eQTL-related genes in Hanwoo backfat. Ontology indicates the functional category, GO Term presents the GO ID and name, and Genes lists the top 10 mapped genes for each term, with the rest shown as “(+n more)”.

Functional Enrichment	Gene Ontology (GO) Term	Genes (Truncated)	False Discovery Rate (FDR)	*p*-Value
Biological Process	GO:0030029 ~ actin filament-based process	*PLS1*, *ECT2*, *MARCKSL1*, *MYO1B*, *VIL1*, *ARHGEF10L*, *NEB*, *XIRP2*, *KANK4*, *ESPNL* (+97 more)	2.48939 × 10^−7^	1.07394 × 10^−10^
Biological Process	GO:0061061 ~ muscle structure development	*DES*, *SPEG*, *NEB*, *TMOD4*, *MYF6*, *MYF5*, *LMOD2*, *PDLIM5*, *PGM5*, *TMOD1* (+24 more)	2.75832 × 10^−7^	3.56988 × 10^−10^
Biological Process	GO:0031032 ~ actomyosin structure organization	*ECT2*, *ARHGEF10L*, *NEB*, *TMOD4*, *LMOD2*, *LIMCH1*, *PGM5*, *TMOD1*, *ROCK2*, *RTKN* (+14 more)	0.000136244	7.57241 × 10^−7^
Biological Process	GO:0007169 ~ transmembrane receptor protein tyrosine kinase signaling pathway	*EPHA3*, *EPHB1*, *EPHB3*, *CBLB*, *FGR*, *NRP2*, *ERBB4*, *PID1*, *EPHA4*, *IGFBP5* (+60 more)	0.000136244	5.73298 × 10^−7^
Cellular Component	GO:0030017 ~ sarcomere	*SYNC*, *DES*, *NEB*, *FHL3*, *TMOD4*, *PPP1R12A*, *LMOD2*, *MYOZ2*, *PDLIM5*, *TMOD1* (+28 more)	5.4 × 10^−13^	3 × 10^−15^
Cellular Component	GO:0016459 ~ myosin complex	*MYH15*, *MYL1*, *MYO1B*, *MYO1A*, *MYO9B*, *MYO1F*, *MYO6*, *MYO1E*, *MYO5C*, *BMF* (+19 more)	3.10439 × 10^−8^	4.77598 × 10^−10^
Cellular Component	GO:0005925 ~ focal adhesion	*LPP*, *ITGB2*, *LIMS2*, *ASAP3*, *ITGB6*, *XIRP2*, *PARVB*, *PARVG*, *ARHGAP24*, *PTK2B* (+9 more)	0.001318107	4.05571 × 10^−5^
Molecular Function	GO:0003774 ~ cytoskeletal motor activity	*MYH15*, *DNAH7*, *MYO1B*, *KIF5C*, *KIF1A*, *MYO1A*, *DNAH11*, *CENPE*, *KIF24*, *KIF13B* (+38 more)	1.29268 × 10^−8^	4.5601 × 10^−11^
Molecular Function	GO:0019199 ~ transmembrane receptor protein kinase activity	*EPHA3*, *EPHB1*, *EPHB3*, *NRP2*, *ERBB4*, *EPHA4*, *ACVR1*, *ACVR1C*, *DDR2*, *STYK1* (+18 more)	0.000891088	7.02566 × 10^−6^
Molecular Function	GO:0005261 ~ monoatomic cation channel activity	*KCNAB1*, *KCNH8*, *CATSPER4*, *CHRND*, *CHRNG*, *SCN3A*, *SCN9A*, *CHRNA1*, *SCN7A*, *KCNJ13* (+65 more)	0.003135404	3.7081 × 10^−5^
Molecular Function	GO:0005201 ~ extracellular matrix structural constituent	*COL6A5*, *COL6A6*, *COL4A4*, *COL4A3*, *COL11A1*, *COL1A2*, *MMRN1*, *MEGF9*, *COL5A1*, *LAMA2* (+6 more)	0.00667519	1.20924 × 10^−4^
Molecular Function	GO:0004683 ~ calmodulin-dependent protein kinase activity	*MKNK1*, *CAMK2B*, *CAMK2D*, *CAMK4*, *CAMK2A*, *CAMK1D*, *MAPKAPK2*, *MAPKAPK3*, *CAMK2G*, *EEF2K* (+1 more)	0.00527478	6.93138 × 10^−5^

## Data Availability

The sequencing data generated in this study are publicly available in ArrayExpress under accession E-MTAB-13398 and can be downloaded from the European Nucleotide Archive (ENA; Project PRJEB67523, Run Accessions: ERR12122059–ERR12122063 and related entries).

## Supplementary Materials

The following supporting information can be downloaded at https://www.mdpi.com/article/10.3390/ani15213082/s1. Supplementary Table S1. Complete list of significant cis-eQTLs (within 100 kb), including statistics for each SNP-gene pair (β ± SE, effect direction, t-statistic, explained variance, and 95% confidence interval). Supplementary Table S2. List of trans-eQTL hotspots defined by the 99th percentile cutoff (≥10 genes per SNP); 147 SNPs regulating more than ten genes are summarized. Supplementary Table S3. Genes regulated by SNPs 6:60512276 and 21:17035557, including associated statistics for each target gene. Supplementary Table S4. Summary of explained variance by eGene, presenting the lead SNP per gene and its corresponding statistics. Supplementary Table S5. Statistically significant trans-eQTL hotspots confirmed by 100,000 permutations (FDR q < 0.05).
